# Morphology of juvenile stages of *Kuschelina bergi* (Harold) with biological information (Coleoptera, Chrysomelidae, Alticini)

**DOI:** 10.3897/zookeys.561.5950

**Published:** 2016-02-08

**Authors:** Nora Cabrera, Alejandro Sosa, Marta Telesnicki, Mic Julien

**Affiliations:** 1 División Entomología, Facultad de Ciencias Naturales y Museo, La Plata, Paseo del Bosque s/n, 1900 La Plata, Argentina; 2 Fundación para el Estudio de Especies Invasivas-FUEDEI, Bolivar 1559 (B1686EFA) Hurlingham, Buenos Aires, Argentina; 3 IFEVA, Facultad de Agronomía, Universidad de Buenos Aires, CONICET, Av San Martín 4453 (C1417DSE) Buenos Aires, Argentina; 4 CSIRO Ecosystem Science & Biosecurity Flagship, GPO Box 2583, Brisbane, 4001, Australia

**Keywords:** Galerucinae, Alticini, flea beetle, biological control, Phyla, Argentina

## Abstract

*Kuschelina bergi* (Harold, 1881) is being studied to be evaluated as a natural enemy of Phyla nodiflora var. minor (Hook.) N. O’Leary & Múlgura (Verbenaceae), an invasive weed in Australia. Eggs, and 1^st^ and 3^rd^ instar larvae are described and illustrated for the first time. The following characters distinguish *Kuschelina bergi*: presence of two medial setae in prosternum, mesosternum and metasternum, absence of tubercle on sternum I and eight setae in abdominal segment IX. The 3^rd^ instar larvae of *Kuschelina bergi* resemble *Kuschelina gibbitarsa* (Say) larvae: the body shape and details of mouthparts are similar, but the morphology of the mandible is different, as is the tarsungulus which has a single seta. Differences between *Kuschelina bergi* and other known larvae of Oedionychina are discussed. New biological data based on laboratory rearing and field observation are also presented and discussed.

## Introduction

*Kuschelina* Bechyné (1951) is a genus of Alticini leaf beetles, included in the subtribe Oedionychina (Scherer 1983, Duckett and Kjer 2003). It comprises approximately 30 species mainly distributed in temperate and subtropical areas of South America: Paraguay, Perú, Bolivia, Brazil, Chile, Uruguay and Argentina, and another seven species mentioned from Mexico and USA (Scherer 1983). Information on immature stages and on the biology and host–plants of *Kuschelina* is scarce. Some species were collected on Lamiaceae and Scrophulariaceae (Jolivet and Hawkeswood 1995).

*Kuschelina bergi* was originally described by Harold (1881) in the genus *Oedionychis* Latreille, 1829 from Buenos Aires (Argentina) and Montevideo (Uruguay). Since then, only one collection record of this species has been available (Bechyné 1957). This species is associated with the invasive weed, Phyla nodiflora var. minor (Hook.) N. O’Leary & Múlgura 2012 (= *Phyla canescens* (Kunth) Greene) (Verbenaceae). Preliminary observations suggest that this flea beetle could be used as a biological control agent of *Phyla nodiflora* in Australia (Julien et al 2012).

Larval morphology of oedionychines is poorly known. The only detailed study of members of this subtribe are those dealing with the larvae of *Alagoasa parana* Samuelson (1985), *Alagoasa januaria* Bechyné (Duckett and Swigonová 2002), and *Walterianella bucki* (Duckett and Cassari 2002). Mature larva of *Kuschelina gibbitarsa* (Say) were described briefly by Blake (1927), Böving and Craighead (1931), Lawson (1991).

The identification of Kuschelina bergi larvae is important to complete our knowledge of characters in addition to those of the adults and as a first step towards a better understanding of the insect-host plant relationships, needed to conduct studies for biocontrol of Phyla nodiflora var. minor. The purpose of this paper is to describe the immature stages of *Kuschelina bergi*. Biological notes, including field and laboratory host range, are presented as well as some comparative notes on larvae of other Oedionychina species.

## Materials and methods

### Insect collection and study material

Specimens utilized for the study came from adults and larvae of *Kuschelina bergi* which were collected on *Phyla nodiflora* from different locations in Argentina during field trips made from 2009 to 2013. A laboratory colony was set up to obtain sufficient specimens for morphological studies and laboratory tests. Specimens were preserved in 70% ethyl alcohol. The larvae were macerated in 10% KOH solution for several minutes and rinsed in water. The methods of dissection, preparation and examination of immature stages follow Goulet (1977) and May (1979).

Terminology for morphological features of immatures follows Le Sage (1986) and LeSage and Zmudzinska-Krzesinska (2004). Larvae were mounted on metal stubs and coated with gold-palladium and examined with a scanning electron microscope (SEM) Jeol-JSM-T100. Drawings were made using a *camera lucida* on a Leitz compound microscope and a Wild dissecting microscope.

### Biological studies

Biological observations were made on 50 eggs laid by adults collected near Tres Arroyos city (RN 3, Km 520, 25 km S Tres Arroyos S 38°32, W 60°31, Buenos Aires Province) in October 2009 and brought to FuEDEI facilities in Hurlingham, Buenos Aires. They were kept in Petri dishes (10 cm diameter, 2 cm high). The hatched larvae were fed fresh leaves of Phyla nodiflora var. minor. After about 50 days, when the larvae decreased their activity during the prepupal stage, they were transferred to another container (8 cm diameter, 5 cm high) with soil as a substrate for pupation.

Preliminary studies revealed that pupation is a vulnerable stage in the life cycle of *Kuschelina bergi*. Hence, we studied what role the type of substrate and humidity plays on the pupation success through a factorial design experiment. Third instar larvae (N = 180) were collected from the laboratory colony and individually placed in small plastic containers with three different substrates: sand, commercial soil and nothing (as a control) with two levels of humidity (with and without humidity). Treatment with humidity consisted of adding water to containers, at least twice a day, for a completely wet, but not soaking, substrate; treatment without humidity received no water. In the case of the no-substrate test, a piece of tissue paper soaked in water was added for the treatment with humidity. The successful pupation was estimated by registering the emergence of a live adult (yes or no). The data were analyzed using generalized linear modeling, a logistic regression method with over-dispersion accounted for through the use of quasi-binomial error variances. Analysis was performed using R version 3.1.2 (R Core team 2013).

Voucher specimens are deposited in the collections of the Entomology Department, Museo de La Plata (MLPA), Argentina and Fundación para el Estudio de Especies Invasivas Collection FuEDEI, Buenos Aires, Argentina.

## Results

### Description of immature stages of *Kuschelina bergi* (Harold, 1881) (Figures 2–12)

**Figure 1–2.**
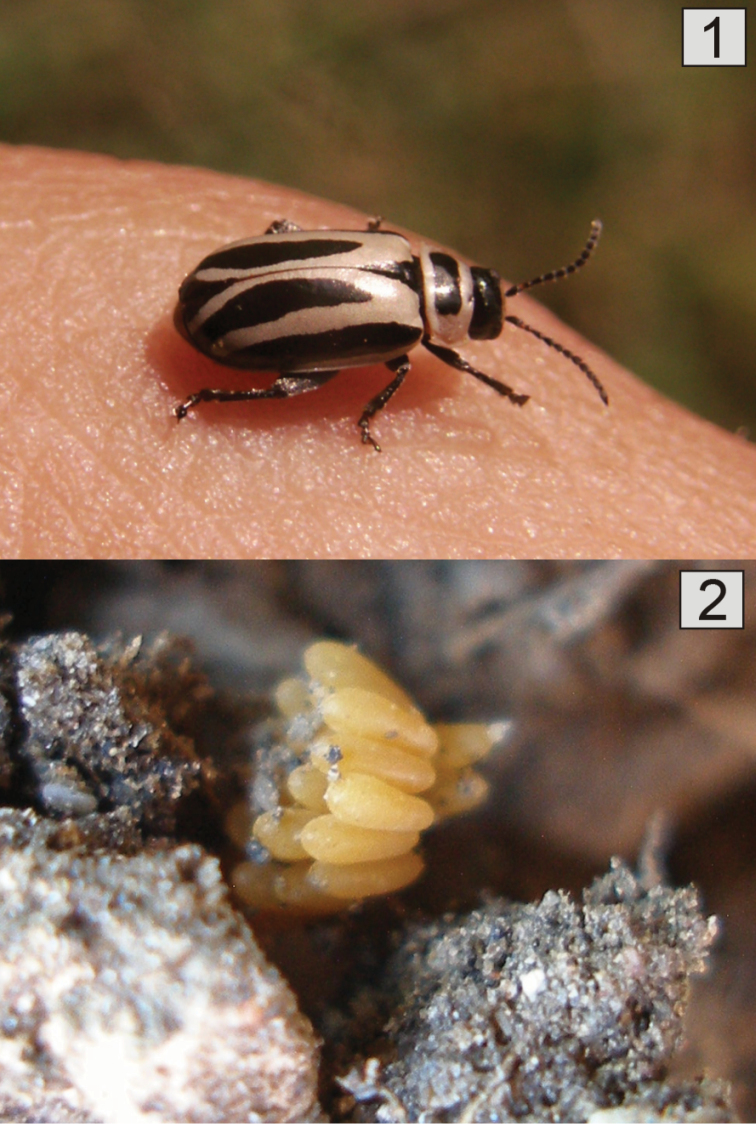
**1** *Kuschelina bergi* (Harold), male, dorsal habitus **2** *Kuschelina bergi* (Harold), cluster of eggs deposited in soil.

**Figure 3–8.**
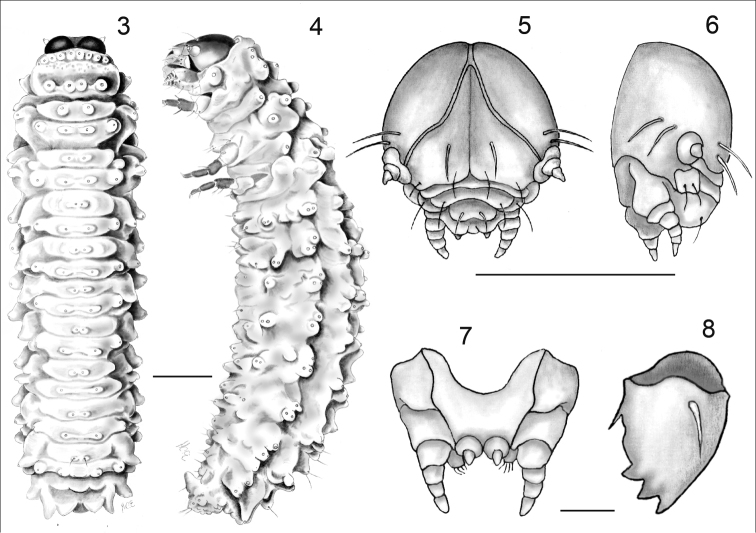
*Kuschelina bergi* (Harold) mature larvae **3** habitus, dorsal view **4** habitus, lateral view **5** cephalic capsule, frontal view **6** cephalic capsule, lateral view **7** labium and maxilla **8** mandible, dorsal view. Scale bars = 1 mm.

**Figure 9–12.**
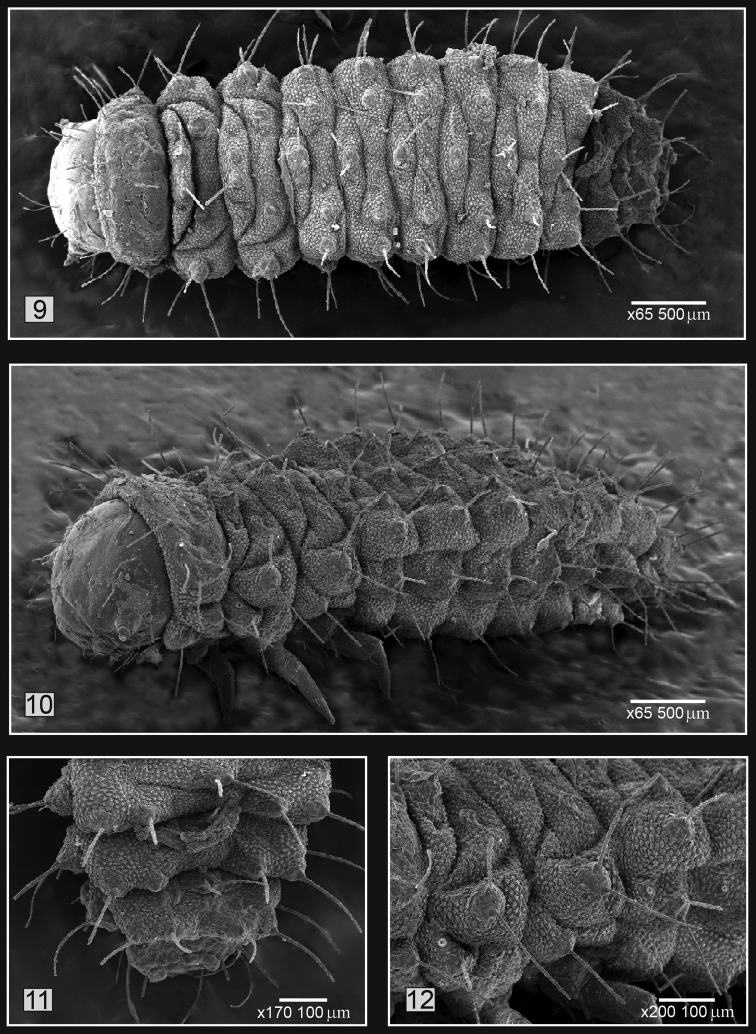
*Kuschelina bergi* (Harold) mature larvae **9** habitus, dorsal view **10** habitus, lateral view **11** abdomen: detail of segments 6-8, dorsal view **12** epipleural area, detail of seta. Scale bars = 100 μm.

**Eggs** (Fig. 2) Shape cylindrical, symmetrical. Coloration bright yellow, surface with fine reticulate impressions. Eggs are laid in clusters or singly, standing on the substrate on one end.

**Mature larvae** (Figs 3–4, 9–10). Length 3.4–3.9mm, body elongate, bright yellow, weakly convex in preserved specimens. Head capsule width 0.5-0.7mm, mouthparts tibiae and tarsi light brown. Thorax yellow tinged with brown.

*Head* (Figs 5–6). Hypognathous, rounded, well sclerotized. Epicranial suture distinct, Y-shaped; well developed, coronal suture short, extending less than one quarter of the length of the head, frontal arms pale, curved, extending to the antennal sockets. Each side of epicranial plate bearing three long setae fixed in the middle of the disc, two above antennal margin and another on the outer side of antennae; numerous micro-setae irregularly distributed. Endocarina present as a black line extending to fronto-clypeal suture. Frons bearing one pair of long setae inserted on the disc, two pairs fixed on anterior margin and another two pairs on clypeal area. Stemmata absent. Antennae short, 3-segmented; attached to head capsule by a large, translucent membrane; basal segment large, transverse; segment II, transverse bearing three setae, with a peg-like sensilla dorsally; segment III formed by a large conical sensory papilla at bearing three long and two sensilla with a large seta fixed on the outer side of antennae. Clypeus transverse, bearing four setae on each lateral margin. Labrum transverse, apical margin emarginated; bearing four large setae in a median row. Mandibles (Fig. 8) robust, slightly sclerotized at apex, 4-toothed; tooth I small, blunt; teeth II-IV subequal, sharply pointed, a long mandibular seta inserted dorso-laterally, penicillus consisting of two thick setae. Maxillae and cardo well-sclerotized, subtriangular, bearing two setae at outer side. Stipes quadrate, with a short, narrow inner seta and two long outer setae, mala densely setose, with numerous short setae. Maxillary palpi with palpiger bearing two long setae; segment I with two short setae; segment III conical. Labium (Fig. 7) with submentum not very sclerotized, widened at base, with a pair of long, filiform submental setae; prementum broad, with a pair of long setae between labial palpi; labial palpi 3-segmented, short. Hypopharynx densely setose.

*Thorax*. Pronotum transverse, bearing two transverse rows of nine anterior and six posterior unisetose tubercles, two fixed at the posterior outer corner; post epipleural tubercle bearing one seta; pre-hypopleural tubercle bearing 2 setae; prosternum with two pairs of median setae. Meso- metathorax subequal, each bearing two anterior and four posterior unisetose tubercles arranged in two rows; mesopleura with alar tubercle bearing two setae; spiracular sclerite bearing one large seta; metapleura with alar tubercle bearing two setae and anterior epipleural sclerite bearing a seta; meso-and metasterna each bearing two median bisetose tubercles. Spiracle annuliform situated in the mesothoracic region. Legs 5-segmented, slightly chitinized, equal in size; trochantin triangular, asetose; coxa trapezoidal bearing eight long setae, two club-like, the others simple, and two sensillae; trochanter, triangular, with four long simple setae; femur sub-rectangular bearing eight long setae; tibia bearing three setae; tarsungulus curved, bearing a setiform pulvillus with a short, thin seta.

*Abdomen*. Abdominal segments I-VIII with filiform setae, dorsally arranged into two rows, interior prescutal area bearing two setae, posteriorly interior scutoscutellar area with two setae, exterior scutoscutellar with one seta and posterior parascutal areas bearing one seta; epipleural area (Fig. 12) bearing two setae; ventrally segments I-VIII with a eusternellar area bearing two setae, interior sternellar areas each with two setae; exterior sternellar bearing two setae; segment IX (Fig. 11) forming a fleshy pygopod with eight pairs of long, filiform setae. Spiracles I-VIII annuliform, situated on the pleural tubercles.

**First instar larva.** The first instar larva is very similar to the mature larva, but smaller in size, length 1.0–1.3mm, head capsule width 0.2–0.5mm. Recently emerged first instars are pale yellow. First instars can be distinguished from the mature larvae by the following characters: each side of epicranial plate with three long setae fixed in the middle of the disc, two above antennal margin and another on the outer side of antennae. Frons bearing one pair of long setae inserted on the disc, two pairs fixed on anterior margin and another two pairs on clypeal area. Egg bursters conical, sclerotized, situated on exterior scutoscutellar sclerites of meso-and metathorax.

**Material examined. ARGENTINA: Buenos Aires**: 13♂♂, RN 3, Km 520, 25 km S Tres Arroyos S 38°32, W 60°31, Sosa col.; 1♂, RP 11, Nueva Atlantis, S36°85 W56°69, Sosa col. (FUEDEI); 8♀♀, Rt. 226, 1 Km. E Bolívar, S36°26 W61°69, Sosa col. (FUEDEI).

**Biological aspects.** In the field, adults (Fig. 1) were found in leaf and stem litter on the ground. Adults and larvae feed on leaves; the former make circular holes, first instar larvae just scratch the epidermis and the final instars feed from the edge of the leaves towards the center.

Females dig a hole under the plants and lay eggs in masses of approximately ten eggs. Three larval instars were recorded, and the complete larval development took about two months. Pupation occurred in the soil and lasted over two weeks.

**Figure 13.**
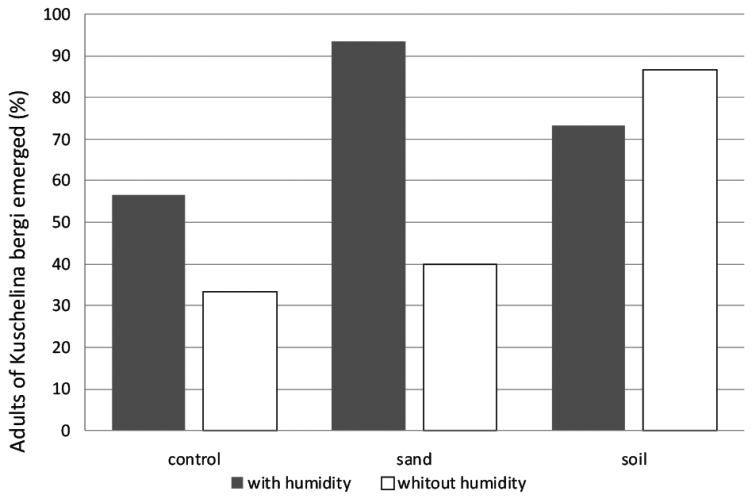
Adults of *Kuschelina bergi* emerged from three different kind of substrate in two conditions of humidity.

In the laboratory, the rate of emergence of adult *Kuschelina bergi* varied depending on the type of substrate and the humidity (Fig. 13). The pupation study revealed that *Kuschelina bergi* makes a “pupation chamber” with material from the substrate. In the dry sand containers, the larvae were covered in sand particles but they were not able to bury themselves nor were they able to build the chambers.

Pupation was more successful in moist sand, similar results were obtained in moist soil (t = -2.845, P = 0.00498), and dry soil showed slightly better results than moist soil.

In the “no substrate” treatment, where tissue paper was added for the tests with humidity, very few adults were able to emerge and survival was very low. Where there was no substrate and no moisture, again, a very low number of adults were able to emerge and the survival was extremely low. These results will strongly help to improve rearing methods.

## Discussion

The brief description of *Kuschelina gibbitarsa* precludes detailed comparisons with *Kuschelina bergi* but both species share the following features: body shape, mouthparts with large mala carrying long setae along inner margin, submentum and mentum are fused. However, in contrast with the membranous pulvillus illustrated by Lawson (1991), we observed that *Kuschelina bergi* only have a single seta on the inner margin of the tarsungulus, and the mandible has a pyramidal shape. *Kuschelina bergi* can be recognized by the presence of pro-meso-and metasterna with two medial setae, eight dorsal setae in abdominal segment 9 and the absence of tubercle on sternum I.

Duckett and Swigoňová (2002) listed morphological characters of the known Oedionychinae larvae (*Walterianella bucki*, *Alagoasa parana*, *Alagoasa januaria*, and *Kuschelina gibbitarsa*) and mentioned that the prominent tubercles, chaetotaxy and the absence of stemmata are the main characters for recognizing larvae of the Oedionychina. Herein we summarized (Table 1) the diagnostic characters of *Kuschelina bergi* to facilitate comparison with the other larvae studied by Duckett and Swigoňová.

**Table 1.** Morphological comparison of third instar larvae of Walterianella bucki Bechyné, Alagoasa parana Samuelson, Alagoasa januaria Bechyné, Kuschelina gibbitarsa (Say), and Kuschelina bergi (Harold) (modified from Duckett and Swigoňová 2002).

Third instar	*Walterianella bucki*	*Alagoasa parana*	*Alagoasa januaria*	*Alagoasa gibbitarsa*	*Kuschelina bergi*
Club-like setae	present	absent	absent	absent	absent
Prominency of tubercles	dorso-lateral only	All dorsal and dorso-lateral	dorso-lateral only	dorso-lateral only	dorso-lateral only
Dorsolateral tubercles	Prominent and large	Less prominent, short and broad	Less prominent, long and thin	Less prominent, short and broad	Less prominent, short and broad
Median setae on prosternum	1 pair	1 pair	2 pairs	Not described	1 pair
Ventro-lateral tubercle of sg. I	present	Not present	present	Not described	Not present
Tubercles on meso-and metanotun	2 posterior unisetose	4 posterior unisetose	4 posterior unisetose	4 posterior unisetose	4 posterior unisetose
Dorsolateral tubercle sg. I-VIII	I-VII unisetose, VIII bisetose	bisetose	unisetose	Bi or trisetose	bisetose
Setation abd. sg. IX	8 dorsal, 6ventral setae	Not described	8dorsal,8 ventral setae	12 dorsal,10 ventral setae	8 dorsal setae
**First instar**					
Egg bursters	Absent	Present	Present	Present	Present

### Final considerations

The status of *Kuschelina* as currently defined is somewhat unclear (Scherer 1983, Duckett 1998, Duckett and Swigonová 2002). Adults of some species are confused with mimetic forms of *Alagoasa*, a widely distributed genus in South and Central America. The first phylogenetic analysis of the subtribe Oedionychina (Duckett and Kjer 2003) suggested a close relationship between species of both genera.

As few larvae have been studied so far, we cannot make generalizations about not only the value of the characters to determine taxa but also for phylogenetic studies. Detailed descriptions (adult and immature stages) of *Kuschelina* are much needed and will contribute to resolving the taxonomic relationships among species and will provide biological knowledge on the Alticini, especially within the subtribe Oedionychina.
